# Enhancing Postoperative Analgesia After Cesarean Section: Insights Into Transversus Abdominis Plane Block, Intrathecal Opioids, and Other Analgesic Techniques

**DOI:** 10.7759/cureus.72773

**Published:** 2024-10-31

**Authors:** Salah N El-Tallawy, Joseph V Pergolizzi, Haneen F Amlih, Moaaz M Fairaq, Fouad I Awaleh, Abdullah T Alsubaie, Issam S Shaheen, Yusra S Al-Kayyal, Rania S Ahmed, Wegdan A Ali

**Affiliations:** 1 Anesthesia, King Khalid University Hospital, College of Medicine, King Saud University, Riyadh, SAU; 2 Anesthesia and Pain Management, Faculty of Medicine, Cairo University, Cairo, EGY; 3 Anesthesia and Pain Management, Faculty of Medicine, Minia University, Minia, EGY; 4 Research, NEMA Research, Inc., Naples, USA; 5 Obstetrics and Gynecology, College of Medicine, Alfaisal University, Riyadh, SAU; 6 Anesthesia, Faculty of Medicine, Minia University, Minia, EGY

**Keywords:** cesarean section, general anesthesia, neuraxial analgesia, postoperative pain, spinal anesthesia, spinal opioids, transversus abdominis plan block

## Abstract

Background: Cesarean section (CS) is associated with moderate to severe pain that may delay recovery and interfere with the mother’s ability to take care of the newborn. This clinical study aimed to evaluate different analgesic protocols for postoperative pain management after CS.

Methods: The study included 300 parturients scheduled for CS and classified into five equal groups as follows: general anesthesia (GA group), GA plus transversus abdominis plane (TAP) block (GA+TAP group), spinal anesthesia (SA) plus intrathecal fentanyl (ITF group), SA plus fentanyl + TAP block (ITF+TAP group), and SA plus intrathecal morphine (ITM group). Multimodal analgesia was added to all groups in the form of paracetamol 1 g/6 hours intravenously (IV), lornoxicam (8 mg/8 hours/IV), in addition to rescue opioid analgesia (morphine or oxycodone 4 mg/IV) upon the patient’s request. Primary outcomes included pain assessments by numerical rating scale (NRS), worst and least pain scores, and time in maximum pain. Secondary outcomes included time to first request of analgesia, total opioid consumption, patient request for additional analgesia, function, and satisfaction scores. Possible side effects and neonatal Apgar scores were recorded at five and 10 minutes.

Results: The results showed a significant difference between the study groups regarding pain relief. The best pain relief, lowest worst pain, least pain, and total opioid consumption were reported by the ITM group, followed by ITF+TAP, GA+TAP, then SA, and GA groups (p < 0.05). The lower percentage of time in the worst pain and the longest time to the first request of analgesia were reported with ITM and ITF+TAP groups followed by the ITF group and GA+TAP group. In contrast, the longest time spent in the worst pain and the shortest duration until the first request of analgesia was observed in the GA group (p < 0.05). The best function scores were observed with ITF+TAP and then ITM, while the highest satisfaction scores were reported with ITM, ITF+TAP followed by SA and GA+TAP, and the lowest scores were observed in the GA group (p < 0.001 and p < 0.005, respectively). Side effects such as itching, nausea, and vomiting were significantly higher in the ITM group when compared to the other groups (p < 0.05). There were no significant differences between the study groups regarding the Apgar scores at five and 10 minutes (p = 0.271 and 0.760).

Conclusions:Bilateral TAP block has the potential to effectively enhance postoperative analgesia and reduce the need for systemic opioids after both GA and SA. Compared to ITF, ITM provides a higher quality and longer duration of analgesia with minimal analgesic-related side effects. Further studies to find an optimal analgesic protocol with few to no side effects are warranted.

## Introduction

Cesarean section (CS) is one of the most frequently performed surgeries in the world. It is associated with moderate to severe postoperative pain, especially during the first 24 hours [1,2]. Inadequately controlled postoperative pain may delay the mother’s recovery, impede breastfeeding, and interfere with maternal-neonatal bonding [3,4]. Effective postoperative analgesia is an important factor in improving clinical outcomes, enhancing tissue healing, and restoring maternal function [5]. Ideal postoperative pain relief after CS has benefits for the mother including successful feeding, minimal drug transfer through breast milk, and increased maternal satisfaction [6]. The choice of the anesthetic technique is the most important factor in determining the overall outcomes, especially for postoperative pain [5,6]. The PROSPECT recommendation for postoperative analgesia after CS has established a multimodal pre-, intra-, and postoperative analgesic strategy, which, combined with certain surgical approaches and adjuvant techniques, may provide excellent analgesia [4].

The anesthetic technique is exclusively neuraxial anesthesia [7] although spinal anesthesia (SA) is recognized as the technique of choice for both elective and emergency CS. The main disadvantage of single-shot SA is the absence of long-lasting postoperative analgesia, which may necessitate the administration of additional analgesic medications postoperatively. Thus, neuraxial opioids such as intrathecal fentanyl or morphine can be added to the local anesthetics to enhance the quality of SA and prolong the duration of postoperative analgesia [8,9]. Moreover, regional analgesia is strongly recommended to improve the quality of analgesia and enhance recovery as a part of an enhanced recovery after surgery (ERAS) protocol [10]. Several regional techniques can be used, such as local anesthetic wound perfusion, liposomal bupivacaine, and fascial plane blocks [7]. We hypothesized that the fascial plane block and/or spinal opioids might augment the postoperative analgesia after CS when they are added to the anesthetic/analgesic techniques. This study aims to evaluate the efficiency and efficacy of different analgesic protocols for postoperative pain relief after CS and to determine the most suitable regimen that provides optimal pain relief with rapid recovery while minimizing adverse effects.

## Materials and methods

Study design

**Figure 1:**
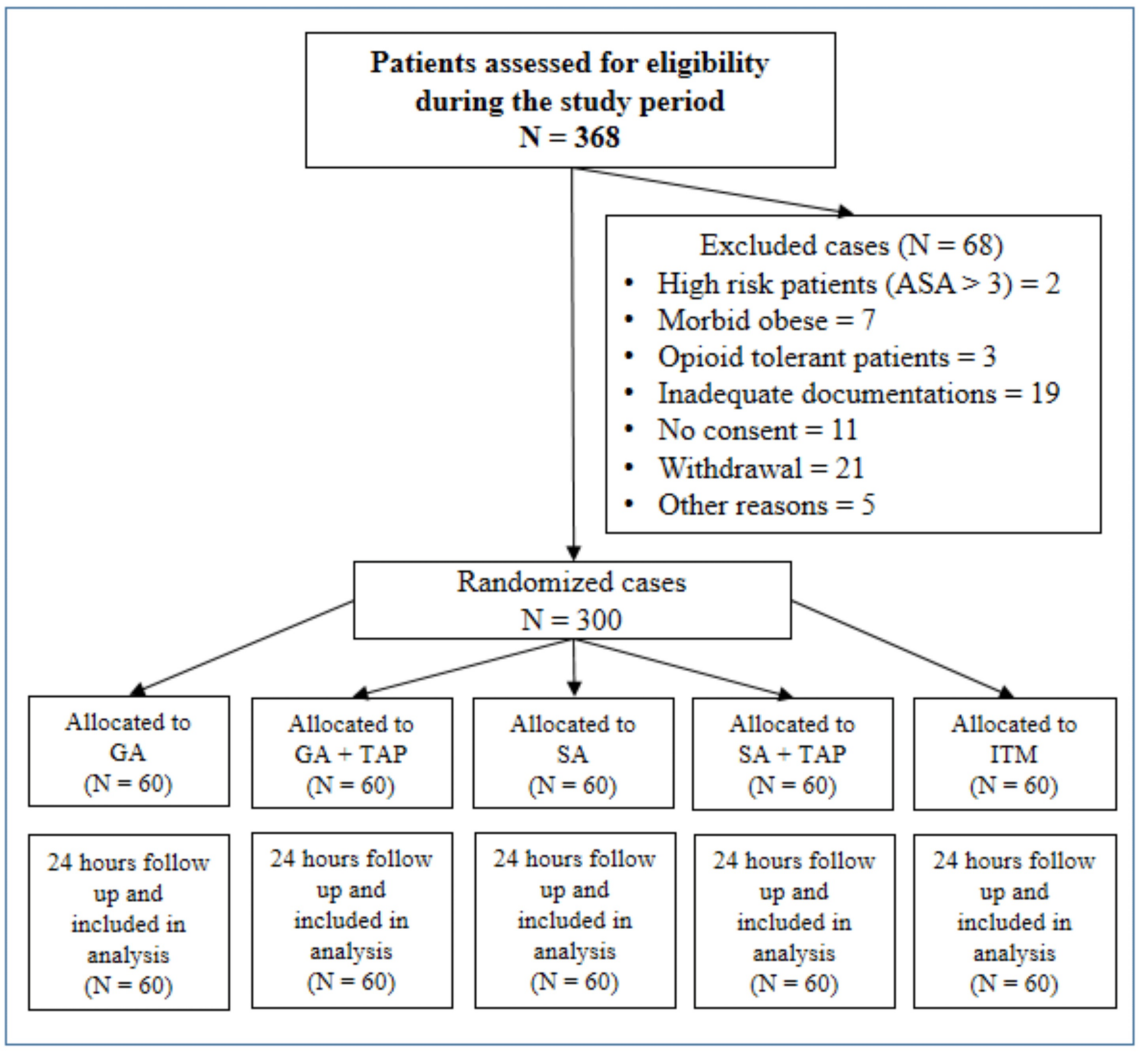
CONSORT flow chart of the patients included into this study CONSORT: Consolidated Standards of Reporting Trials; ASA: American Society of Anesthesiologists; GA: general anesthesia; TAP: transversus abdominis plane block; SA: spinal anesthesia plus fentanyl; ITM: spinal anesthesia plus morphine

The data from this randomized, double-blinded clinical trial were collected from one center at King Khalid University Hospital, King Saud University, Saudi Arabia, as part of a quality improvement plan for improving postoperative pain outcomes. Parturients who underwent elective or emergency CS under general (GA) or spinal anesthesia (SA) between 2019 and 2022 were randomly enrolled in this study. The study was approved by the Institutional Review Board (IRB) of the College of Medicine, King Saud University, Riyadh, Saudi Arabia (Ref. No. 18/0443/IRB). The trial was registered before patient enrollment at clinicaltrials.gov (NCT05624502, registration date: January 1, 2019). Trained nurses from the acute pain services (APSs) or research assistants, blinded to the treatment groups and not involved in the anesthetic technique, approached the patients on the ward on the first postoperative day during the routine rounds of the APS team. The anesthetic technique, the method of postoperative pain relief, and the pain assessment tools were explained to all patients by the anesthesia team during the routine preoperative assessments. The written consent form for being included in the quality improvement program was obtained from all patients on the next postoperative day by the APS team during the daily pain round. Patients then reported their pain scores and side effects related to pain therapy after CS. Per the protocol, data were collected from all patients on the first postoperative day. The Consolidated Standards of Reporting Trials (CONSORT) statement was followed to report this randomized controlled trial (Figure 1) [11].

Participants

Inclusion criteria included the following: full-term pregnant women, scheduled for elective or emergency CS, their age between 18 and 40 years, American Society of Anesthesiologists (ASA) physical status I or II, willingness to participate, and the ability to provide informed consent. Exclusion criteria included the following: patient refusal, known allergy to the study medications, known contraindication to the anesthetic or analgesic techniques, history of opioid dependence or drug abuse, significant comorbidities that could affect pain perception or analgesic requirements (e.g., severe cardiac, pulmonary, liver, renal impairment, or coagulation disorders), and associated obstetric problems (e.g., multiple gestations, antepartum hemorrhage, or fetal distress).

Outcome measures

Primary Outcomes

The numerical rating scale (NRS) ranging from 0 (no pain) to 10 (worst possible pain) was used for pain assessment. The pain scores were measured at rest and during movements (i.e., pain that interferes with normal activities in and out of bed) over the first 24 hours. The minimum and maximum pain scores along with the duration of time spent in maximum pain, were also recorded over the first 24 hours.

Secondary Outcomes

They included the time to the first analgesic request for rescue opioid analgesia and the total consumption of rescue opioids (e.g., morphine or oxycodone) during the first 24 hours postoperatively. The patient’s request for additional analgesia due to uncontrolled pain was also recorded.

Functional Score

This simple score utilizes functional ability to determine pain relief. The three-point functional activity score has been developed as the following [12]: (A) refers to no limitation of (relevant) activity because of pain, (B) mild limitation of activity because of pain, and (C) the patient is unable to complete activity because of pain. The overall maternal satisfaction with postoperative pain management was assessed using a score from 0 to 10. Neonatal outcomes, including Apgar scores measured at five and ten minutes were recorded. The incidence of opioid-related side effects (e.g., sedation, respiratory depression, nausea, vomiting, and pruritus) was also recorded.

Group assignment and analgesic interventions

A total of 300 participants were enrolled in this study. The patients were divided into five equal groups. A sealed envelope was used for randomization. One of the obstetric anesthesia team opened the envelope 30 minutes before the procedure. The participants were equally assigned to one of the following five groups based on the anesthetic technique and postoperative analgesic protocols. (1) GA group (N = 60): Patients who received GA with multimodal analgesia consisting of paracetamol 1 g IV/6 hours, lornoxicam 8 mg IV/8 hours, and opioid boluses (e.g., morphine or oxycodone 4 mg IV). (2) GA plus transversus abdominis plane (GA+TAP block group) (N = 60): Patients who received GA with a postoperative TAP block along with multimodal analgesia in the form of paracetamol 1 g IV/6 hours, lornoxicam 8 mg IV/8 hours, and opioid boluses (e.g., morphine or oxycodone 4 mg IV). (3) ITF group (N = 60): Patients who received intrathecal anesthesia using 10 mg bupivacaine and 25 mcg intrathecal fentanyl, along with multimodal analgesia consisting of paracetamol 1 g IV/6 hours, lornoxicam 8 mg iv/8 hours, and opioid boluses (e.g., morphine or oxycodone 4 mg IV). (4) ITF+TAP block group (N = 60): Patients who received intrathecal anesthesia using 10 mg bupivacaine and 25 mcg intrathecal fentanyl with a postoperative TAP block, along with multimodal analgesia consisting of paracetamol 1 g IV/6 hours, lornoxicam 8 mg IV/8 hours, and opioid boluses (e.g., morphine or oxycodone 4 mg IV). (5) ITM group (N = 60): Patients who received intrathecal anesthesia using 10 mg bupivacaine and 100 mcg intrathecal morphine along with multimodal analgesia in the form of paracetamol 1 g IV/6 hours, lornoxicam 8 mg iv/8 hours, and opioid boluses (e.g., morphine or oxycodone 4 mg IV).

Study procedures

Preoperative Assessment

The preoperative assessment was conducted for all patients as a standard protocol before surgery. Baseline assessments, including demographic data, medical history, and obstetric history were collected. Routine laboratory tests and coagulation profiles were performed for all patients. A history of chronic pain, use of opioids, or other controlled medications over the last few months was also assessed.

Anesthetic Techniques

The anesthetic management and intraoperative care were conducted by the assigned obstetric anesthesia team. Standard anesthetic protocols were followed for all participants, including SA or GA, per institutional guidelines. Preoperative assessments, with basic and specific laboratory tests when required, were performed for all participants before they were transferred to the operative room. The standard monitors were connected. Noninvasive monitors, including heart rate, arterial blood pressure, electrocardiography (ECG), oxygen saturation, and end-tidal CO₂, were continuously monitored in the operating room. After the delivery of the baby, all patients received dexamethasone (8 mg IV), paracetamol (1 g IV), and lornoxicam (8 mg IV). After surgery, patients were transferred to the postanesthesia care unit (PACU) for routine follow-up for at least four hours according to the hospital policy.

SA groups: Pre- or co-loading with 500 ml Ringer’s lactate was done for all patients. In the operating room, while parturients were in a sitting position and under strict aseptic technique, they received SA with a 25-27G atraumatic spinal needle. A dose of 10 mg of 0.5% hyperbaric bupivacaine plus 25 mcg fentanyl in the ITF group or 100 mcg morphine in the ITM group was injected after negative aspiration for the blood.

GA groups: A balanced GA was given to all patients scheduled for GA. Standard monitors were connected before induction of the anesthesia. GA was induced by propofol (1.5-2.5 mg/kg/IV), skeletal muscle relaxant rocuronium (0.5 mg/kg/IV) was given to facilitate endotracheal intubation, and the inhalational anesthetic sevoflurane was maintained between 1.5% and 3%). After delivery of the baby, fentanyl (2-3 mcg/kg/IV) was given to the patient. After surgery, neuromuscular blockade was reversed by neostigmine (2.5 mg/IV) with glycopyrrolate (0.4 mg/IV). Patients were transferred to the PACU for routine follow-up for at least two hours.

TAP block: This was conducted only in the fascia plane block groups (GA+TAP and ITF+TAP groups). After surgery, while the patients were still anesthetized, a bilateral TAP block was performed. Complete sterile precautions were strictly followed. The linear high-frequency ultrasound probe was placed at the midaxillary line, between the costal margin and the iliac crest, with an imaging depth of 4-5 cm, using the in-plane technique, the needle was inserted a few centimeters medial to the probe until the tip reached between the internal oblique and the transversus abdominis muscles. Hydro-dissection was done with 1-2 mL saline to confirm the proper needle position between the fascial layers. After confirming the correct localization, a total volume of 20 ml of the 0.25% bupivacaine was injected on each side. During and after the block, the parturient was monitored for any signs of local anesthetic toxicity. Following the block, patients were transferred to the PACU.

Postoperative Pain Management

Postoperative pain assessments were conducted by the APS team during their daily pain round. It is a part of the quality improvement program to improve postoperative pain outcomes. The APS team was blinded to the group assignment and the study design. They collected the data from all patients as per the quality improvement project and the study protocol. Postoperative pain intensity scores at rest and during movements were assessed at regular intervals (e.g., baseline pain score at the PACU and then at one, three, six, 12, and 24 hours) using the NRS. The minimum pain score, maximum pain score, and time spent in maximum pain as reported by each patient during the first 24 hours, were also recorded. These scores were recorded by trained personnel from the APSs blinded to the treatment groups.

Postoperative opioid analgesia: The total amount of rescue opioid consumption within the first 24 hours postoperatively was recorded. The time to the first request of analgesia representing the duration of time from recovery to the first request for rescue analgesia was also recorded.

Quality of Pain Management

Function score: Scott and McDonald [12] developed this three-point functional activity score as follows: (A) No limitation of (relevant) activity because of pain, (B) mild limitation of activity because of pain, and (C) unable to complete activity because of pain. Maternal satisfaction with pain management was assessed using a validated questionnaire with a score ranging from 0 to 10. A request for additional analgesia due to unrelieved pain was documented. Neonatal outcomes using the Apgar scores were recorded at five and ten minutes for all newborns.

Adverse events: The occurrence of opioid-related side effects (e.g., nausea, vomiting, pruritus, excessive sedation, or respiratory depression) was recorded. Any other adverse events or complications related to the analgesic protocols were recorded.

Ethical considerations

This study is part of a quality improvement program aimed at enhancing postoperative pain management. The study was conducted using ethical guidelines and regulations. Informed consent was obtained from all participants before inclusion in the quality project. A detailed description of the study’s purpose, procedures, potential risks, benefits, and participants' rights was recorded. Patient confidentiality and privacy were maintained throughout the project. Patients had the right to withdraw their consent at any time during the study. Any adverse events or complications were promptly reported and managed appropriately.

Sample size calculation

A power analysis was performed to determine the required sample size based on the expected effect size, significance level, and statistical power. The statistical power was calculated before enrolling patients. The number of patients was estimated based on an expected reduction in pain intensity measured by (NRS) of 20%. The estimated minimal sample size was 120 patients, with a power of 80% and a significance level of 5%. The number of included patients was increased to compensate for incomplete data. Additionally, as previously mentioned, the study was a part of the quality improvement plan, with a large number of patients enrolled over the three-year study duration (2019 to 2022). All study participants met the study inclusion criteria.

Data collection and statistical analysis

Data were collected from all patients regularly during the first postoperative day. IBM SPSS Statistics for Windows, Version 27 (Released 2020; IBM Corp., Armonk, New York, United States) was used for statistical analysis. Statistical analyses were performed using appropriate methods. Data were expressed as means + SD or number and percentage according to the type of data. Descriptive statistics were applied to summarize the demographic and clinical characteristics of the participants. The primary and secondary outcomes were analyzed using appropriate statistical tests. Unpaired t-tests were used to compare two groups for parametric data. The Mann-Whitney U test was applied for a nonparametric alternative to the unpaired t-test. A one-way analysis of variance (ANOVA) test was applied to compare the outcomes between more than two groups for parametric data. If significant differences were found, posthoc tests (e.g., Tukey's test) were conducted to identify specific group differences. The Kruskal-Wallis test was applied to compare nonparametric data for more than two groups. In case significant differences are found, posthoc tests (e.g., Dunn's posthoc test) can be conducted to identify specific group differences. The chi-square test or Fisher's exact test was used to compare the ASA physical status distribution, the percentage of time in severe pain, and the incidence of side effects among the different analgesic groups. A significant result was considered when p < 0.05.

## Results

Demographic characteristics

The study included 300 participants who underwent CS under either GA or SA. Subsequently, based on the type of postoperative analgesia, the patients were divided into five groups (N = 60 women per group): GA, GA+TAP, ITF, ITF+TAP, and ITM. All patients received multimodal analgesia for postoperative pain relief in the form of paracetamol (1 g IV/6 hours), nonsteroidal anti-inflammatory drugs (NSAIDs) (meloxicam 8 mg/IV/8 hours), and rescue opioid analgesia (morphine or oxycodone) 4 mg IV/4 hours as needed. The demographic characteristics of the included patients are shown in Table 1. There were no significant differences between the study groups regarding demographic data (p > 0.05).

**Table 1:** Patient characteristics GA group: General anesthesia group; GA+TAP group: general anesthesia plus transversus abdominis plane (TAP) block group; ITF group: spinal anesthesia (SA) plus intrathecal fentanyl group; ITF+TAP group: SA plus fentanyl + TAP block group; ITM group: SA plus intrathecal morphine ITM group; BMI: Body mass index; ASA: American Society of Anesthesiologist Data were shown as mean + SD (e.g., age and BMI) or number and percentage (e.g., ASA, parity, and type of CS). (*) Comparison between the five groups regarding age and BMI was done using an ANOVA test. (**) Comparison between the five groups regarding ASA physical status, parity, and type of CS was done using chi-square test

	GA group	GA+TAP group	ITF group	ITF+TAP group	ITM group	p-value
Age (Y)*	23.325 + 3.970	24.275 + 3.835	23.65 + 4.347	24.48 + 3.088	25.02 + 2.96	0.127
BMI (kg/m^2^)*	29.26 + 5.07	28.93 + 4.52	28.32 + 5.25	29.31 + 3.98	30.21 + 2.41	0.197
ASA (N/%)**						
ASA I	33 (55%)	34 (56.66%)	32 (53.33%)	33 (55%)	35 (58.33%)	0.052
ASA II	27 (45%)	26 (44.33%)	28 (46.66%)	27 (45%)	25 (41.66%)	
Parity (N/%)**						
Primigravida	20 (33.33%)	19 (31.66%)	18 (30%)	21 (35%)	18 (30%)	0.240
Multigravida	40 (66.66%)	41 (68.33%)	42 (70%)	39 (65%)	42 (70%)	
Type of CS (N/%)**						
Elective	44 (73.33%)	42 (70%)	45 (75%)	43 (71.66%)	45 (75%)	0.054
Emergency	16 (26.66%)	18 (30%)	15 (25%)	17 (22%)	15 (28.33%)	

Postoperative pain management

Pain Scores

Table 2 shows the postoperative pain scores, assessed using the NRS after CS, both at rest and during movements throughout the first 24 hours post-surgery. A comparison between the five groups by the Kruskal-Wallis test revealed significant differences between the groups at the baseline, and at one, three, 12, and 24 hours after surgery for static pain (p < 0.001, p = 0.002, p = 0.014, p < 0.001, and p = 0.004, respectively). No significant differences in static pain were observed between the study groups at six hours post-surgery (p = 0.31). Regarding the dynamic pain scores, a comparison between the five groups by the Kruskal-Wallis test showed significant differences between the groups at the baseline and at one, three, six, 12, and 24 hours after surgery (p < 0.001, p < 0.001, p < 0.001, p < 0.001, p = 0.008, and p < 0.001, respectively). Dunn's post hoc test showed that the best pain relief was recorded in the ITM group, followed by the ITF+TAP and GA+TAP groups, and then the ITF group. The least effective pain relief was observed in the GA group in most of the readings except the static pain after six hours, where there was no significant difference between the groups (Table 2).

**Table 2:** Postoperative pain scores in the five groups in rest (static) and during movements (dynamic) by NRS GA group: General anesthesia group; GA+TAP group: general anesthesia plus transversus abdominis plane (TAP) block group; ITF group: spinal anesthesia (SA) plus intrathecal fentanyl group; ITF+TAP group: SA plus fentanyl + TAP block group; ITM group: SA plus intrathecal morphine ITM group Data were presented as mean + SD. (*) Comparison between the five groups was done using Kruskal-Wallis test. Dunn's posthoc test showed that pain intensity was significantly less in the ITM group followed by ITF+TAP group, then GA+TAP and ITF groups and the least effect in GA group in all readings except the static pain after six hours where there were no significant differences between the study groups

	GA group	GA+TAP group	ITF group	ITF+TAP group	ITM group	p-value
Static pain baseline	5.75 + 1.58	4.2 + 1.4	2.25 + 1.06	1.55+0.81	2.33+1.185	<0.001
Dynamic pain baseline	7.53 + 1.05	6.75 + 1.79	2.93 + 0.91	1.95+0.71	3.55+1.154	<0.001
Static pain after 1 hour	3.2 + 1.81	3.28 + 1.64	2.26 + 0.92	2.15 + 0.80	2.25 + 1.532	0.002
Dynamic pain after 1 hour	4.5 + 1.30	2.9 + 1.25	3.85 + 1.36	2.38 + 1.12	2.33 + 1.591	<0.001
Static pain after 3 hours	2.68 + 0.917	2.10 + 0.59	2.25 + 0.81	2.15 + 0.53	2.08 + 0.554	0.014
Dynamic pain after 3 hours	4.5+1.30	2.7 + 1.07	2.85 + 0.92	1.96 + 0.69	1.83 + 1.238	<0.001
Static pain after 6 hours	2.05 + 0.50	1.80 + 0.68	1.88 + 0.76	1.65 + 0.62	1.50 + 0.552	0.31
Dynamic pain after 6 hours	4.1 + 0.84	2.23 + 0.89	3.15 + 1.33	1.9 + 0.74	1.50 + 1.194	<0.001
Static pain after 12 hours	2.55+1.01	1.83+0.74	1.80+0.76	1.95+0.90	1.45+1.085	<0.001
Dynamic pain after 12 hours	3.35+1.09	2.63+0.80	2.58+0.91	2.58+0.75	2.25+0.707	0.008
Static pain after 24 hours	2.47+0.75	2.0+0.93	1.82+0.84	2.35+0.86	2.15+0.863	0.004
Dynamic pain after 24 hours	3.6+1.07	3.2+0.79	2.77+1.31	2.55+0.75	1.925+0.859	<0.001

Worst and Least Pain Scores

Regarding the worst pain scores, results showed that the best pain relief was observed in the ITM group followed by the ITF+TAP and GA+TAP groups and then the ITF group, while the least pain relief was observed in the GA group (Table 3) (p < 0.001). The lowest pain scores showed that the best pain relief was observed in the ITM group followed by the ITF+TAP and then ITF and GA+TAP groups, while the least pain relief was observed in the GA group (p < 0.001) (Table 3).

**Table 3:** Worst pain, least pain, and the duration of percentage of time in worst pain during the first 24 hours in the study groups GA group: General anesthesia group; GA+TAP group: general anesthesia plus transversus abdominis plane (TAP) block group; ITF group: spinal anesthesia (SA) plus intrathecal fentanyl group; ITF+TAP group: SA plus fentanyl + TAP block group; ITM group: SA plus intrathecal morphine ITM group; ANOVA: analysis of variance Data were shown as mean + SD, number and percentage (e.g., type of opioid and need for more analgesia), or percentage only (e.g., time in worst pain). Comparison between the five groups was done using a one-way ANOVA test. (*) Comparison by between the five groups was done by Kruskal-Wallis test. (#) Comparison by chi-square test. p < 0.05: statistically significant

	GA group	GA+TAP group	ITF group	ITF+TAP group	ITM group	p-value
Worst pain (NRS from 0 to 10)^*^	6.27 + 1.22	4.87 + 0.87	5.45 + 1.02	4.32 + 0.97	4.275 + 1.012	<0.001
Least pain score^*^	1.67 + 0.85	1.0 + 0.64	0.7 + 0.56	0.47 + 0.38	0.35 + 0.49	<0.001
Time in worst pain (%) ^#^	10-30	10-20	10 – 20	5 - 20	5-20	<0.001
Rescue analgesia (mg/24 hours)	19.60 + 6.675	12.50 + 5.592	11.80 + 6.984	10.50 + 3.863	8.15 + 3.244	<0.001
Type of opioid:						
Morphine	48 (80%)	46 (76.67%)	47 (78.33%)	49 (81.67%)	48 (80%)	0.087
Oxycodone	12 (20%)	14 (23.33%)	13 (21.67%)	11 (18.33%)	12 (20%)	
First request of analgesia (min)	51.75 + 22.97	93.25 + 50.44	119.87 + 50.9	129 + 56.32	140.6 + 51.17	<0.001
Need for more analgesia^#^	26 (43.33%)	16 (26.66%)	14 (23.33%)	9 (15%)	3 (5%)	<0.001
Function score^*^	1.52 + 0.64	1.97 + 0.63	1.92 + 0.66	2.27 + 0.67	2.13 + 0.667	<0.001
Satisfaction score^*^	5.25 + 1.66	5.9 + 1.61	6.05 + 1.63	6.18 + 1.708	6.75 + 1.9	0.005
Apgar score^*^	8.55 + .815	8.475 + 0.784	8.40 + 0.90	8.40 + 0.84	8.50 + 0.93	0.271
Apgar score^*^	9.03 + 0.32	9.14 + 0.45	9.30 + 0.72	9.070 + 0.28	9.50 + 0.13	0.760

The percentage of time spent in worst pain was significantly of shorter duration in the ITM and ITF+TAP groups followed by the SA and GA+TAP groups, while the longest time spent in worst pain was observed in the GA group (p < 0.001) (Table 3). The time to the first request of analgesia was significantly longer in the ITM group, followed by the ITF+TAP group, then the ITF and GA+TAP groups. In contrast, the shortest time to the first request of analgesia was observed in the GA group (p < 0.001) (Table 3). Rescue opioid analgesic consumption and the percentage of patients who required additional analgesia during the first 24 hours were higher in the GA group followed by the GA+TAP group, then the ITF group, while the lowest consumption and the need for more analgesia were observed in the ITM and ITF+TAP groups (Table 3). A comparison of opioid consumption by ANOVA test and the frequency of analgesic request was done using the chi-square test (p < 0.001 both)

Quality of pain management

Function Scores

The highest function scores were observed in the ITF+TAP followed by the ITM, then GA+TAP, and ITF, and the lowest function score was observed with the GA group. A comparison of the function scores was done using the Kruskal-Wallis test which showed significant differences between the groups (p < 0.001).

Satisfaction Scores

The best patient satisfaction scores were observed in ITM and then ITF+TAP groups, followed by ITF and GA+TAP groups, while the lowest scores were observed in the GA group (Table 3). A comparison of function scores was done using the Kruskal-Wallis test which showed significant differences between the groups (p < 0.005).

Apgar Scores

Apgar scores at five and 10 minutes showed no significant differences between the study groups. A comparison was done using the Kruskal-Wallis test (p = 0.271 and p = 0.760, respectively).

Comparison between individual groups

Comparison between GA and GA+TAP and between ITF and ITF+TAP groups regarding worst pain, least pain, percentage of time spent in worst pain, need for more analgesia, function score and patient’s satisfaction time by Mann-Whitney U test showed significant differences in all parameters in favor of TAP block groups. Additionally, a comparison between the same groups regarding the time to the first request of opioid analgesia and opioid consumption by unpaired t-test showed improved outcomes in favor of the TAP groups (Table 4). A comparison between the same groups regarding the need for additional analgesia was conducted using the chi-square test and showed significant differences in favor of the TAP groups (Table 4). Comparison between the GA and ITF groups showed significant improvement in favor of spinal anesthesia except for patient satisfaction (p = 0.116), where this was no significant difference (Table 4). Comparison between GA+TAP versus ITF+TAP groups showed significant improvements in the worst pain, least pain, time to the first request of analgesia, and function scores. No significant differences were found between the two groups regarding the other parameters (Table 4). Comparison between the ITF and ITM groups showed better analgesia in favor of the ITM group regarding the worst pain score, time spent in the worst pain, time to the first request to analgesia, and total opioid consumption during the first 24 hours. The function score was slightly but not significantly better in the ITF group compared to the ITM group (p = 0.053). Moreover, the overall patient satisfaction was in favor of the ITM group compared with the ITF group (p = 0.046) (Table 4).

**Table 4:** Comparison between the individual groups GA group: General anesthesia group; GA+TAP group: general anesthesia plus transversus abdominis plane (TAP) block group; ITF group: spinal anesthesia (SA) plus intrathecal fentanyl group; ITF+TAP group: SA plus fentanyl + TAP block group; ITM group: SA plus intrathecal morphine ITM group Data were presented as mean + SD. For the parametric data (e.g., time in worst pain, time to first request of analgesia and opioid consumption), the comparisons between each two groups were done using unpaired t-test. For the nonparametric data (e.g., worst pain, least pain, satisfaction, and function score), comparison between each two groups were done using the Mann-Whitney U test

Groups	Worst pain	Least pain	Time in worst pain	First request of analgesia	Rescue opioids	Wish for more analgesia	Satisfaction	Function score
GA # GA+TAP	0.006	<0.001	0.01	<0.001	0.025	<0.001	0.044	0.008
ITF # ITF+TAP	0.01	0.03	0.618	0.670	0.307	0.002	0.035	0.045
GA # SA	0.001	0.001	<0.001	<0.001	<0.001	<0.001	0.116	0.003
GA+TAP # ITF+TAP	<0.001	<0.001	0.525	0.034	0.376	<0.001	0.074	0.012
ITF # ITM	0.026	0.013	0.042	<0.001	0.003	<0.001	0.046	0.053

Side effects

Side effects such as itching, nausea, and vomiting were reported only in a few patients among the first four groups, while the ITM group showed a significantly higher incidence of these side effects (e.g. itching, nausea, and vomiting) in comparison to the other groups (P = 0.0015, 0.0023, and 0.006) respectively. No cases of excessive sedation or respiratory depression were reported, and no further interventions were needed for side effects in any of the groups (Table 5). 

**Table 5:** Side effects GA group: General anesthesia group; GA+TAP group: general anesthesia plus transversus abdominis plane (TAP) block group; ITF group: spinal anesthesia (SA) plus intrathecal fentanyl group; ITF+TAP group: SA plus fentanyl + TAP block group; ITM group: SA plus intrathecal morphine ITM group Data were presented as numbers and percentages. Comparison between the five groups was done using chi-square test

	GA group	GA+TAP group	ITF group	ITF+TAP group	ITM group	p-value
Itching	5 (10%) 0-2	6 (12%) 0-1	6 (12%) 0-1	7 (14%) 0-2	18 (28.33%)	0.0015
Nausea	9 (18%) 0-3	8 (16%) 0-1	9 (18%) 0-2	10 (20%) 0-2	14 (23.33%)	0.0023
Vomiting	3 (6%) 0-3	4 (8%) 0-3	3 (6%) 1-3	4 (8%) 0-2	11 (18.33%)	0.006

## Discussion

Postoperative pain following CS is a challenging clinical scenario due to its implications for both the mother and the infant. Therefore, optimizing postoperative analgesia is crucial to promoting enhanced recovery following CS. Poorly managed postoperative pain can not only reduce patient satisfaction but may also increase maternal morbidities and impair a mother's ability to care for her infant [4,13]. Various anesthetic and analgesic techniques exist to improve the quality and prolong the duration of analgesia after CS.

This study aimed to evaluate the efficiency of different analgesic modalities for postoperative pain relief after CS. Five different modalities were compared: GA, GA+TAP block, ITF with intrathecal fentanyl, ITF+TAP block, and SA with ITM. In all groups, multimodal analgesia was given to all patients regularly (e.g., paracetamol and NSAIDs) or when requested by the patients (e.g., rescue opioid boluses). Multimodal analgesic therapy is delivered by intentionally targeting various pain pathways with the overall goal of reinforcing the analgesic effect. Results of the study revealed that TAP block significantly enhances the level of analgesia in the postoperative period when added to either GA or ITF. However, intrathecal morphine gives superior analgesia compared to the other groups and for a longer duration, along with some drawbacks.

The choice of the anesthetic technique (GA versus SA) for CS often depends on maternal and fetal conditions. Previous studies confirm the increasing preference for SA over GA in planned CS procedures [14,15]. Furthermore, GA is associated with longer recovery periods, which can affect postoperatively for both mothers and infants. SA is associated with faster recovery and reduced nausea and vomiting, as it typically requires fewer systemic opioids, which aligns with the increasing preference for this technique [14,15]. Numerous studies have demonstrated that SA offers advantages and better outcomes over GA in parturients undergoing CS [16,17]. These include higher patient satisfaction, shorter hospital stay, faster recovery time, and fewer complications such as sore throat, drowsiness, nausea, and vomiting which were significantly lower with SA compared to GA after CS [16,17]. Furthermore, SA offers other advantages over GA: better control of acute postoperative pain, quicker maternal recovery, facilitation of immediate breastfeeding, and observation of ERAS guidelines [17]. Similar findings showed that neuraxial anesthesia is the method of choice for CS because it is associated with lower morbidity, making it safer for both the neonate and the mother [18,19].

TAP block efficacy

TAP block after CS has the potential to effectively relieve postoperative pain and reduce the need for additional analgesics [20,21]. This argument that TAP block enhances the effectiveness and quality of acute postoperative pain relief after both GA and SA has been well-established [20]. Our findings align with the majority of the literature, which highlights the advantages of incorporating a TAP block after CS to enhance acute postoperative pain relief and improve patient recovery and outcomes [20,21]. This is primarily because TAP block selectively relieves the pain around the anterior abdominal wall as well as the parietal peritoneum which are the predominant sources of pain after CS [22]. In this way, it reduces the use of systemic opioids after CS, whereby a patient’s postoperative recovery period is improved in terms of faster mobility and early mother-neonate contact. The use of TAP block in addition to GA or SA assures higher levels of pain relief. The combination of several modalities adheres to the principle of multimodal analgesia. The effect of TAP for controlling acute postoperative pain decreases after the first 12 hours after surgery emphasizing the importance of an effective multimodal approach [22]. However, this relatively short-term pain relief promotes early mother-infant bonding and facilitates breastfeeding [23].

TAP Block Optimization to Enhance the Quality of Analgesia

There are different approaches to improving the quality of analgesia produced by the TAP block. Data from a meta-analysis demonstrated that a more posterior injection site may produce superior and prolonged analgesia compared with lateral TAP blockade [24]. Recently, a novel technique using liposomal bupivacaine in TAP blocks provided prolonged analgesia following CS, even when combined with ITM [25].

TAP Block Versus Other Peripheral Neural Blockades

Various options for peripheral nerve blocks can be utilized to provide postoperative analgesia after CS [25]. One approach receiving more consideration to provide analgesia after CS is the use of ilioinguinal and iliohypogastric (IL/IH) nerve blocks. A meta-analysis of five studies compared the analgesic effects of TAP and IL/IH nerve blocks. Findings showed no significant differences regarding the time to first rescue analgesic request, total postoperative tramadol consumption, and postpain scores at various time points, both at rest and during movement [26]. The ultrasound-guided transversalis fascia plane block (TFPB) selectively blocks the T12 and L1 dermatomes and can also provide analgesia for lower abdominal surgeries, including CS [27]. A recent systematic review and meta-analysis compared TFPB to placebo (for CS) and other blocks such as quadratus lumborum block (QLB) for postoperative analgesia after lower abdominal surgeries. This meta-analysis found that TFPB was more effective than placebo and comparable to QLB in reducing opioid consumption, extending the time to rescue analgesia, and reducing the incidence of PONV in lower abdominal surgeries, particularly CS [28]. Another systematic review assessed the efficacy of QLB compared to control, neuraxial morphine, or TAP blocks. Results indicated that QLB significantly improved analgesia following CS compared to the control group but provided no additional analgesic benefit when combined with ITM. However, insufficient data were available to make a direct comparison between QLB and TAP blocks [29]. However, another network meta-analysis compared the efficacy of QLB with TAP blocks and found that, in the absence of IT opioids, these two blocks were consistently better than the control group at reducing pain scores and systemic opioid consumption after CS. There were no significant differences in the analgesic effects between TAP and QLB techniques [30].

Intrathecal Opioids (ITF Versus ITM) 

Our results revealed that spinal opioids provide superior pain relief compared to GA alone. The study also demonstrated that intrathecal morphine provides more effective analgesia, in most cases than GA, GA plus TAP block, and even intrathecal fentanyl. Intrathecal morphine remains the most commonly used intrathecal opioid, and it is the only opioid approved by the US Food and Drug Administration for intrathecal administration [31]. The use of intrathecal morphine is more effective in the management of postoperative pain than other systemic opioids or even intrathecal fentanyl. The use of intrathecal morphine is characterized by prolonged analgesia lasting 20-48 hours after various surgical procedures. It is considered the gold standard analgesic technique for Cesarean delivery, which is generally performed under SA [31]. Several earlier studies emphasized its role in lasting postsurgical pain management. According to previous studies, intrathecal morphine provides superior analgesia compared to TAP blocks up to 24 hours after CS [22]. Intrathecal morphine is preferred in comparison to local anesthetic-based RA techniques such as TAP and wound infiltration. A dose of 50 mcg to less than 150 mcg ITM is recommended for maintaining a balance between analgesia and side effects [31]. A meta-analysis comparing low-dose (50-100 mcg) and high-dose (>100-250 mcg) intrathecal morphine for CS found that higher dose was associated with more PONV but no difference in pain scores at 12 and 24 h and no difference in opioid consumption at 24 h [32].

Comparison between intrathecal fentanyl versus intrathecal morphine showed that patients in the morphine group had significantly better pain management and higher levels of satisfaction. This is attributed to the greater need for rescue medication in the fentanyl group. On the other hand, fentanyl had significantly fewer side effects, such as pruritus, nausea, vomiting, and dizziness [33]. Similar results reported greater time elapsing before the need for additional analgesics in the case of morphine [34,35]. Regarding the duration of effective analgesia, previous studies showed that the use of fentanyl in combination with a local anesthetic had about a 12 h effect, whereas the average duration of effective analgesia when ITM in a dose of (100 µg) was 18 to 22 h in similar studies [34,36].

Enhancing the Quality of Analgesia and Its Implication in Clinical Practice

The use of intrathecal morphine for postoperative pain management after CS might not be optimal for intraoperative analgesia. However, although intrathecal fentanyl is beneficial in the early postoperative period, it seems not to be optimal for long-lasting analgesia. Therefore, the combination of intrathecal fentanyl and intrathecal morphine provides better early perioperative analgesia than morphine alone with the associated prolonged analgesia compared to intrathecal fentanyl alone. This technique may be recommended in situations where the time from induction of anesthesia to skin incision is short [37].

Side effects showed that no patients in the study groups developed intolerable side effects that required active intervention or reversal of the opioid action. These results were similar to the findings of a recent systematic review by Grape et al. [38], who reported that intrathecal morphine in a dose of 100 mg represents a threshold dose for nausea and vomiting and does not produce respiratory depression while providing satisfactory analgesia. In contrast to our results, this systematic review showed that intrathecal lipophilic opioids like fentanyl were associated with pruritus [38].

Strengths and limitations

There are several strengths in this study. It included an adequate sample size of 300 parturients. They were classified into five equal groups for a comprehensive comparison of different analgesic techniques. The use of multimodal analgesia (e.g., paracetamol, NSAIDs, opioids, and regional anesthetic techniques) across all the study groups reflects standard clinical practice and helps reduce potential bias. Both the primary and secondary outcomes were assessed, incorporating multimodal pain assessments (static and dynamic pain; highest and lowest pain scores; time spent in worst pain; time to the first analgesic request and total opioid consumption). Additionally, quality indicators for postoperative pain relief such as function scores and patient satisfaction were recorded. These comprehensive measures ensure that the study’s outcomes have practical implications for improving postoperative pain management after CS.

While this study provides valuable insights, several limitations must be acknowledged. Although the sample size is adequate, it may limit the generalizability of the results to broader populations. Additionally, the use of unidimensional pain scores (e.g., NRS), along with the subjective nature of pain assessment, may introduce variability and potential bias. Furthermore, the duration of the study was limited to 24 hours only. A long-term follow-up assessing pain outcomes and patient satisfaction would provide a better understanding of the lasting impacts of these analgesic strategies and could reduce the risk of persistent postoperative pain.

## Conclusions

Postoperative pain relief following CS is extremely important to optimize maternal and neonate well-being. The addition of paracetamol and NSAIDs as a part of multimodal management has the potential to improve the quality of analgesia while reducing opioid requirements. The bilateral TAP blocks should be considered in patients undergoing GA or ITF to improve the quality and duration of postoperative analgesia as well as reduce opioid consumption, particularly in situations where neuraxial anesthesia is not recommended. Intrathecal opioids are the cornerstone for postoperative analgesia in patients undergoing SA. Spinal opioids provide a therapeutic window for effective pain relief using lower doses of opioids, with minimal side effects. ITM offers better postoperative analgesia compared to fentanyl with fewer analgesic-related side effects such as itching, nausea, and vomiting. Despite these side effects, the findings of this study suggest that ITM offers superior postoperative analgesia with longer-lasting analgesia and higher patient satisfaction, making it an excellent option for postoperative pain management following CS. Further studies should be conducted to evaluate adequate doses and ideal mixtures of intrathecal opioids (e.g., fentanyl plus morphine) to provide optimal analgesia with little or no side effects.
